# Impact of bivalirudin on mortality and bleeding complications in acute coronary syndrome patients undergoing invasive revascularization: A real world experience

**DOI:** 10.1007/s00508-016-1078-6

**Published:** 2016-09-13

**Authors:** Miklos Rohla, Ioannis Tentzeris, Matthias K. Freynhofer, Serdar Farhan, Rudolf Jarai, Florian Egger, Thomas W. Weiss, Johann Wojta, Alexander Geppert, Adnan Kastrati, Gregg W. Stone, Kurt Huber

**Affiliations:** 1 3rd Medical Department, Cardiology and Intensive Care Medicine, Wilhelminenhospital, Montleartstraße 37, 1160 Vienna, Austria; 2 Department of Cardiology, Medical University, Vienna, Austria; 3 Deutsches Herzzentrum, Technische Universität, Munich, Germany; 4 Columbia University Medical Center and the Cardiovascular Research Foundation, New York, USA; 5 Medical School, Sigmund Freud University, Vienna, Austria

**Keywords:** Bivalirudin, Acute coronary syndrome, Percutaneous coronary intervention, Anticoagulation, Heparin

## Abstract

**Background:**

In a retrospective analysis of a prospective single center registry we compared the use of bivalirudin, unfractionated heparin (UFH) monotherapy, UFH + abciximab in 1240 consecutive patients with acute coronary syndrome (ACS) undergoing stent implantation.

**Results:**

Bivalirudin was associated with tendentially reduced in-hospital minor or major bleeding rates compared to UFH monotherapy (5.9 % vs. 9.4 % adjusted odds ratio (OR) 0.82, 95 % confidence interval CI 0.45–1.51, *p* = 0.53) and compared to the pooled UFH group (5.9 % vs. 11.9 %, adjusted OR 0.62, 95 % CI 0.36–1.08, *p* = 0.09) but with significantly lower bleeding hazards compared to UFH + abciximab (5.9 % vs. 16 %, adjusted OR 0.41, 95 % CI 0.22–0.78, *p* < 0.01). After 3 years of follow-up, adjusted cardiovascular mortality rates were similar between all groups, particularly between bivalirudin vs. UFH monotherapy (hazard ratio HR 1.12, 95 % CI 0.58–2.16, *p* = 0.73) and vs. UFH + abciximab (HR 0.91, 95 % CI 0.40–2.10, *p* = 0.83). Acute or subacute stent thrombosis occurred at a rate of 0.8 % with no significant differences between the groups.

**Conclusions:**

This retrospective analysis in a real world situation of medium to high-risk ACS patients undergoing invasive revascularization confirmed the results of most large-scale randomized trials by demonstrating reduced bleeding rates in favor of bivalirudin vs. UFH + GPI but with no significant differences between treatment strategies for long-term all-cause and cardiovascular mortality.

## Introduction

Invasive revascularization with stent implantation is the standard of care for the majority of patients presenting with acute coronary syndrome (ACS) and improves clinical outcomes compared to a more conservative approach [1]. Recent studies have suggested that novel antithrombotic and antiplatelet strategies, namely fondaparinux [2], bivalirudin [3, 4], ticagrelor [5] and rivaroxaban in combination with dual antiplatelet therapy [6] may reduce mortality in ACS patients. In recent large-scale prospective randomized trials, the favorable pharmacological action of bivalirudin translated into lower 30-day major bleeding rates compared to unfractionated heparin (UFH) plus a glycoprotein IIb/IIIa inhibitor (GPI) and also vs. UFH monotherapy but exhibited similar or even worse rates in ischemic events depending on study design, patient selection and concomitant therapy [4, 7–9]. While previous guidelines have led to an increased use of bivalirudin in the USA, bivalirudin is infrequently used in ACS patients undergoing percutaneous coronary interventions (PCI) in western Europe (approximately 10 % of patients) with the exception of some highly specialized centers. Of note, international trials have mainly compared bivalirudin (monotherapy) with UFH + GPI in ACS patients and real world observations vs. UFH monotherapy are scarce [3, 9–11]. Accordingly, we investigated the efficacy and safety of bivalirudin compared to heparin-based strategies in patients with ST-elevation or non-ST-elevation myocardial infarction (STEMI and NSTEMI, respectively) referred for acute angiography who subsequently received PCI and stent implantation.

## Patients and methods

### Study population

In this post hoc analysis of a prospective registry, antithrombotic therapy (as used per the discretion of the interventional cardiologist), cardiovascular risk factors, comorbidities and coronary morphology were evaluated in 1240 consecutive patients with ACS who were referred for PCI with stent implantation between January 2004 and June 2012. We included STEMI patients presenting with ST-segment elevation of 1 mm or more in two or more contiguous leads, as well as NSTEMI patients with elevated troponin I, troponin T or creatine kinase MB levels (CK-MB) above the upper limit of normal and/or ST-segment depression of ≥1 mm. Patients lacking laboratory or electrocardiographic evidence suggestive of high-risk ACS were excluded from this analysis as were patients referred for rescue PCI after thrombolytic therapy, patient records with a missing cause of death (*n* = 4) or patients who were in cardiogenic shock at any time during index hospitalization. In contrast to current practice, PCI was performed by the femoral approach in the vast majority of patients included into the registry (93 %). The study was performed in accordance with the Declaration of Helsinki and approved by the local ethics committee (EK 10-046-VK_NZ).

### Study treatment

As the institutional standard of care, all patients received aspirin and clopidogrel as a 600 mg loading dose (LD) followed by 75 mg maintenance dose (MD), prasugrel (60 mg LD followed by 10 mg MD except in patients >75 years or <60 kg who received 5 mg MD) or ticagrelor (180 mg LD followed by 180 mg MD). Prasugrel was used in most STEMI patients and in high-risk NSTEMI patients starting in 2010. All STEMI patients were pretreated with UFH (60 IU/kg i.v. bolus but maximum 5000 U) by emergency physicians. All NSTEMI patients received anticoagulation with enoxaparin, UFH or fondaparinux until diagnostic coronary angiography. In the case of fondaparinux being given prior to catheterization, UFH was exclusively added in the catheterization laboratory as i.v. bolus of 85 IU/kg body weight. The NSTEMI patients who were pretreated with enoxaparin or UFH and STEMI patients either received UFH in the catheterization laboratory or bivalirudin, which was administered as bolus and infusion only until the end of the intervention due to practical reasons (both STEMI and NSTEMI: 0.75 mg/kg bolus, 1.75 mg/kg/h infusion). If extended bed rest after PCI was indicated, enoxaparin was administered at a thrombosis prophylactic dose until full mobilization. Abciximab was used in the presence of increased thrombus load in the infarct-related artery or as bail-out therapy in patients with slow or no-reflow in the recommended dosage (bolus +infusion for 12 h). Moreover, patients received optimal secondary prevention, i. e. statin therapy with a target low-density lipoprotein (LDL) of less than 70 mg/dl (1.8 mmol/l) but also other guideline-recommended treatment, such as beta blockers, angiotensin converting enzyme (ACE) inhibitors and/or angiotensin receptor blockers. All ACS patients received parenteral anticoagulation, aspirin and an approved P2Y_12_ inhibitor, as early as possible, usually in the emergency room of the department but in the majority before the catheter laboratory. Decisions regarding the choice of P2Y_12_ inhibitor, bail-out use of abciximab, access site, performance of thrombus aspiration, stent type and duration of dual antiplatelet therapy were left to the physician’s discretion.

### Study outcome

The primary efficacy endpoint was cardiovascular mortality after 3 years of follow-up. As secondary efficacy endpoints we evaluated all-cause mortality after 3 years of follow-up as well as acute and subacute stent thrombosis. Mortality data for all patients were obtained from Statistics Austria, an independent and non-profit-making federal institution under public law that supports scientific services. Cases of death occurring in Austria are centrally recorded by Statistics Austria and data are made available for authorized institutions on request. Cause of death is reported by means of the International Statistical Classification of Disease and Related Health Problems (ICD-10). A cardiovascular death was adjudicated when an ICD-10 chapter I00-I99 diagnosis was reported as the cause of death. Acute and subacute stent thrombosis was evaluated and reported according to the definition proposed by the Academic Research Consortium [12]. The primary safety endpoint was a composite of in-hospital minor or major bleeding events based on the Thrombolysis in Myocardial Infarction (TIMI) classification. As tertiary endpoint we registered days from PCI to hospital discharge. This tertiary endpoint was compared between patients with and without bleeding complications and between the different antithrombotic strategies. For the detection of in-hospital bleeding, levels of hemoglobin and hematocrit were measured before and daily after PCI until hospital discharge. For categorization of periprocedural bleeding events, we calculated the difference in hemoglobin concentrations between the most recent hemoglobin value before PCI and the hemoglobin nadir post-PCI. In accordance with the TIMI bleeding classification [13] the following cut-off levels were used: minor bleeding was defined as a drop in hemoglobin from ≥3 to <5 g/dl or a ≥10 % decrease in hematocrit and major bleeding was defined as a drop in hemoglobin ≥5 g/dl or a ≥15 % decrease in hematocrit.

Clinical outcome data were compared between the pooled bivalirudin group (with or without GPI use) vs. UFH monotherapy, vs. UFH + abciximab and vs. the pooled UFH group. As the group of patients receiving bivalirudin + abciximab represented only 1.1 % of the whole cohort (14 patients), a separate analysis for this subgroup was not performed.

### Statistical methods

Descriptive statistics were performed on baseline variables and stratified by the different treatment groups. Discrete characteristics are expressed as frequency counts and percentages and differences were determined by the χ^2^-test. Continuous characteristics are expressed as means and standard deviations or medians (where appropriate), with differences examined with the Kruskal-Wallis test throughout all groups or the Mann-Whitney test for the comparison of two treatment arms. The level of significance used for all tests was a two-sided *p*-value of 0.05.

Univariate analysis in the Cox proportional hazards and the logistic regression model was performed to estimate mortality and bleeding hazards between different antithrombotic treatments. A 1:1 matched propensity score for the abovementioned two-group comparisons was obtained in order to account for confounding variables. Covariates were chosen based on significant differences between groups (Table 1) and well-established risk factors, e.g. age, gender, body mass index (BMI), estimated glomerular filtration rate (eGFR), baseline hemoglobin, hypertension, hyperlipidemia, smoking, diabetes, clinical presentation (STEMI or NSTEMI), type of stent, number of diseased vessels, previous MI, previous invasive revascularization, peripheral artery disease, prior stroke or transient ischemic attack (TIA), heart failure, history of malignancies, atrial fibrillation, vascular access site, year of index hospitalization, statin treatment and antiplatelet substance. The corresponding propensity scores were then included in the Cox regression (mortality) and logistic regression (in-hospital bleeding) analyses for the two-group comparisons.

**Table 1:** Baseline characteristics of patients stratified by antithrombotic treatment groups

		All patients *n* = 1240	Pooled bivalirudin *n* = 287	UFH monotherapy *n* = 596	UHF + GPI *n* = 357	*p*-value*
*Clinical characteristics*						
Gender	Male	66.80 %	64.80 %	63.30 %	74.20 %	0.002
–	Female	33.20 %	35.20 %	36.70 %	25.80 %	–
Clinical presentation	NSTEMI	48.60 %	59.60 %	54.90 %	29.40 %	<0.001
–	STEMI	51.40 %	40.40 %	45.10 %	70.60 %	–
Drug-eluting Stent	drug eluting stent	48.10 %	67.90 %	44.60 %	38.10 %	<0.001
Access site	Femoral	93.30 %	90.90 %	93.10 %	95.50 %	0.067
Age (years, median)		63	63	65	59	<0.001
BMI (median kg/m^2^)		27.17	27.34	27.02	27.34	0.119
eGFR (median ml/min)		88.68	91.61	82.73	96.52	<0.001
Baseline creatinine (median mg/dl)		0.9	0.9	0.97	0.9	0.053
SBP (median mm Hg)		140	140	135	140	0.758
CRP (median mg/l)		4	3.5	4.7	4	0.155
Baseline Hb (median g/dl)		14.15	14.1	13.8	14.5	<0.001
Baseline PLTs (median g/l)		232	230	233	231	0.603
Peak troponin (median ng/ml)^b^		13.97	9.55	9.53	26.62	<0.001
CK-MB (median U/l)		119	93	99.5	170	<0.001
*Cardiovascular risk factors*						
Hypertension		77.50 %	80.80 %	78.00 %	73.90 %	0.105
Hyperlipidaemia		81.40 %	81.20 %	80.40 %	83.20 %	0.553
Smoking (current or prior)		50.80 %	54.00 %	44.30 %	59.10 %	<0.001
Diabetes		26.00 %	30.30 %	27.30 %	20.40 %	0.011
*Comorbidities*						
Previous MCI		15.00 %	14.60 %	16.80 %	12.30 %	0.173
Previous PCI		14.20 %	16.40 %	14.40 %	12.00 %	0.286
Previous CABG		2.90 %	3.50 %	3.20 %	2.00 %	0.440
Prior stroke or TIA		6.40 %	7.00 %	8.10 %	3.10 %	0.009
PAD		5.90 %	5.90 %	7.00 %	3.90 %	0.140
Atrial fibrillation		6.50 %	5.60 %	8.70 %	3.40 %	0.004
History for malignancies		5.30 %	5.60 %	6.70 %	2.80 %	0.033
Heart failure		11.60 %	11.10 %	11.20 %	12.60 %	0.786
*Diseased vessels*						
1-VD (one vessel disease)		52.00 %	50.20 %	52.00 %	53.50 %	0.286
2-VD (two vessel disease)		30.60 %	33.10 %	31.90 %	26.60 %	–
3-VD (three vessel disease)		17.30 %	16.70 %	16.10 %	19.90 %	–
*Medication therapy*						
ARB		13.10 %	15.60 %	11.40 %	13.80 %	0.217
Beta blocker		85.30 %	86.10 %	84.30 %	86.40 %	0.639
Statins		92.40 %	96.50 %	88.40 %	95.80 %	<0.001
Acetylsalicylic acid		99.20 %	99.10 %	98.80 %	100 %	0.191
*Antiplatelet substance*						
Clopidogrel		76.10 %	68.30 %	80.00 %	75.90 %	<0.001
Prasugrel		13.90 %	24.00 %	9.40 %	13.20 %	–
Ticagrelor		2.00 %	4.20 %	1.80 %	0.60 %	–
Other^a^		8.00 %	3.50 %	8.70 %	10.40 %	–

*UFH* unfractionated heparin, *GPI* glycoprotein IIb/IIIa inhibitor, *STEMI* ST elevation myocardial infarction, *NSTEMI* Non-ST elevation myocardial infarction, *BMI* body mass index, *eGFR* estimated glomerular filtration rate, *SBP* systolic blood pressure, *CRP* baseline c‑reactive protein levels, *Hb* haemoglobin, *HCT* baseline haematocrit, *PLT* baseline platelet count, *CK-MB* creatine kinase myocardial band, *MCI* myocardial infarction, *PCI* percutaneous coronary intervention, *CABG* coronary artery bypass graft, *TIA* transient ischemic attack, *PAD* peripheral artery disease, *ARB* angiotensin receptor blocker, *VD* vessel disease

*χ^2^-test/Kruska-Wallis test throughout three groups (pooled bivalirudin, UFH, UFH +GPI)

^a^Other refers to ticlopidin or inclusion in randomized trials with blinded treatment assignment

^b^Upper limit of normal for troponin I was 0.045 ng/ml

Likewise, the impact of bleeding and antithrombotic therapy on the duration of hospitalization was analyzed in a linear regression model. The Software Package for Social Sciences version 22 (SPSS, Chicago, IL) was used for all statistical calculations.

## Results

### Study population

Registered baseline characteristics included cardiovascular risk factors, comorbidities, coronary morphology, medication at hospital discharge and laboratory findings (Table 1). From 1240 patients presenting with ACS and undergoing PCI plus stent implantation, 632 (51.4 %) patients presented with STEMI. Of the total cohort, 273 (22 %) received bivalirudin monotherapy in the catheterization laboratory, 14 (1.1 %) received bivalirudin + abciximab, 596 (48.1 %) received UFH alone and 357 (28.8 %) patients received UFH + abciximab. The NSTEMI patients more frequently received bivalirudin, while STEMI patients more frequently received UFH + abciximab (*p* < 0.01, Table 1). The utilization of the different antithrombotic strategies over time is presented in Fig. 3. There was a steep increase in bivalirudin use, starting from 2009 (*p* for trend <0.01).

Similarities as well as significant differences regarding baseline characteristics were detected between the study groups. While patients receiving UFH monotherapy, as compared to the pooled bivalirudin group were similar with respect to age, diabetes, hypertension, previous stroke, myocardial infarction or PCI, heart failure, coronary morphology and other characteristics (Table 1), these patients had lower levels of hemoglobin at admission (median 14.1 g/dl vs. 13.8 g/dl, *p* < 0.01) and lower levels of estimated glomerular filtration rate (eGFR, median 91.6 ml/min vs. 82.7 ml/min, *p* = 0.01). Patients receiving UFH + abciximab were younger (median age 63 years vs. 59 years, *p* < 0.01), had higher levels of baseline hemoglobin (median 14.1 mg/dl vs. 14.5 mg/dl, *p* < 0.01) and had a higher eGFR than patients of the pooled bivalirudin cohort (median eGFR 91.6 ml/min vs. 96.5 ml/min, *p* < 0.01). Risk factors and comorbidities, such as diabetes, hypertension, prior stroke, TIA or a history for malignant tumors were more prevalent in the pooled bivalirudin group, as opposed to UFH + abciximab. Discharge medications including ACE inhibitors, ARBs and beta blockers were similar between groups, while patients treated with UFH monotherapy received adequate statin therapy at hospital discharge less frequently than patients treated with bivalirudin or with UFH + abciximab (Table 1, *p* < 0.001). Moreover, bivalirudin treated patients received one of the novel P2Y_12_-receptor inhibitors twice as frequently compared to UFH-treated patients. In total, 88.9 % of patients with drug-eluting stent (DES) implantation received dual antiplatelet therapy for 12 months, while 8.7 % were treated for 6–9 months only. Therapy durations that were either shorter or longer were observed in 2.4 % of patients.

### Mortality outcome

Patients were followed for up to 3 years after the index hospitalization. In total, 113 (9.1 %) events occurred. Rates of all-cause death were 8.7 %, 10.2 % and 7.6 % in the pooled bivalirudin, UFH monotherapy and UFH + abciximab groups, respectively. Corresponding rates of cardiovascular death were 4.9 %, 6.4 % and 4.8 % after 3 years of follow-up, respectively. Univariate analysis revealed non-significant different rates of all-cause or cardiovascular death for bivalirudin vs. UFH monotherapy, vs. UFH + abciximab and the pooled UFH group as well as for UFH monotherapy vs. UFH + abciximab (Table 2).

**Table 2:** Unadjusted and adjusted 3‑year outcomes and rates of stent thrombosis

**Univariate outcomes**						
–	*All-cause death*			*CV death*		
	*HR*	*95 % CI*	*p-value*	*HR*	*95 % CI*	*p-value*
Pooled bivalirudin vs. UFH monotherapy	0.84	0.53; 1.34	0.47	0.76	0.41; 1.40	0.38
Pooled bivalirudin vs. UFH +GPI	1.16	0.67; 2.00	0.59	1.04	0.51; 2.10	0.92
Pooled bivalirudin vs. pooled UFH	0.94	0.60; 1.47	0.79	0.85	0.47; 1.52	0.57
UFH vs. UFH +GPI	1.34	0.88; 2.20	0.17	1.36	0.77; 2.41	0.29
**Adjusted outcomes**						
–	*All-cause death*			*CV death*		
	*HR*	*95 % CI*	*p-value*	*HR*	*95 % CI*	*p-value*
Pooled bivalirudin vs. UFH monotherapy	1.12	0.68; 1.84	0.66	1.12	0.58; 2.16	0.73
Pooled bivalirudin vs. UFH +GPI	0.9	0.48; 1.71	0.75	0.91	0.40; 2.10	0.83
Pooled bivalirudin vs. pooled UFH	1.09	0.68; 1.76	0.71	1.08	0.58; 2.01	0.81
UFH monotherapy vs. UFH +GPI	0.76	0.46; 1.25	0.29	0.75	0.40; 1.41	0.37
**Stent thrombosis**						
–	*All*	*Definite*	*Probable*	*Acute*	*Subacute*	*p* = 0.748*
Pooled bivalirudin	0.4 %	0.4 %	0 %	0 %	0.4 %	–
UFH monotherapy	0.8 %	0.5 %	0.3 %	0.3 %	0.5 %	–
UFH +GPI	1.1 %	0.6 %	0.6 %	0.3 %	0.8 %	–

*UFH* unfractionated heparin, *GPI* glycoprotein IIb/IIIa inhibitor, *HR* hazard ratio, *CI* confidence interval, *CV* cardiovascular

*χ^2^-test, multivariate adjustment not performed

**Fig. 1:**
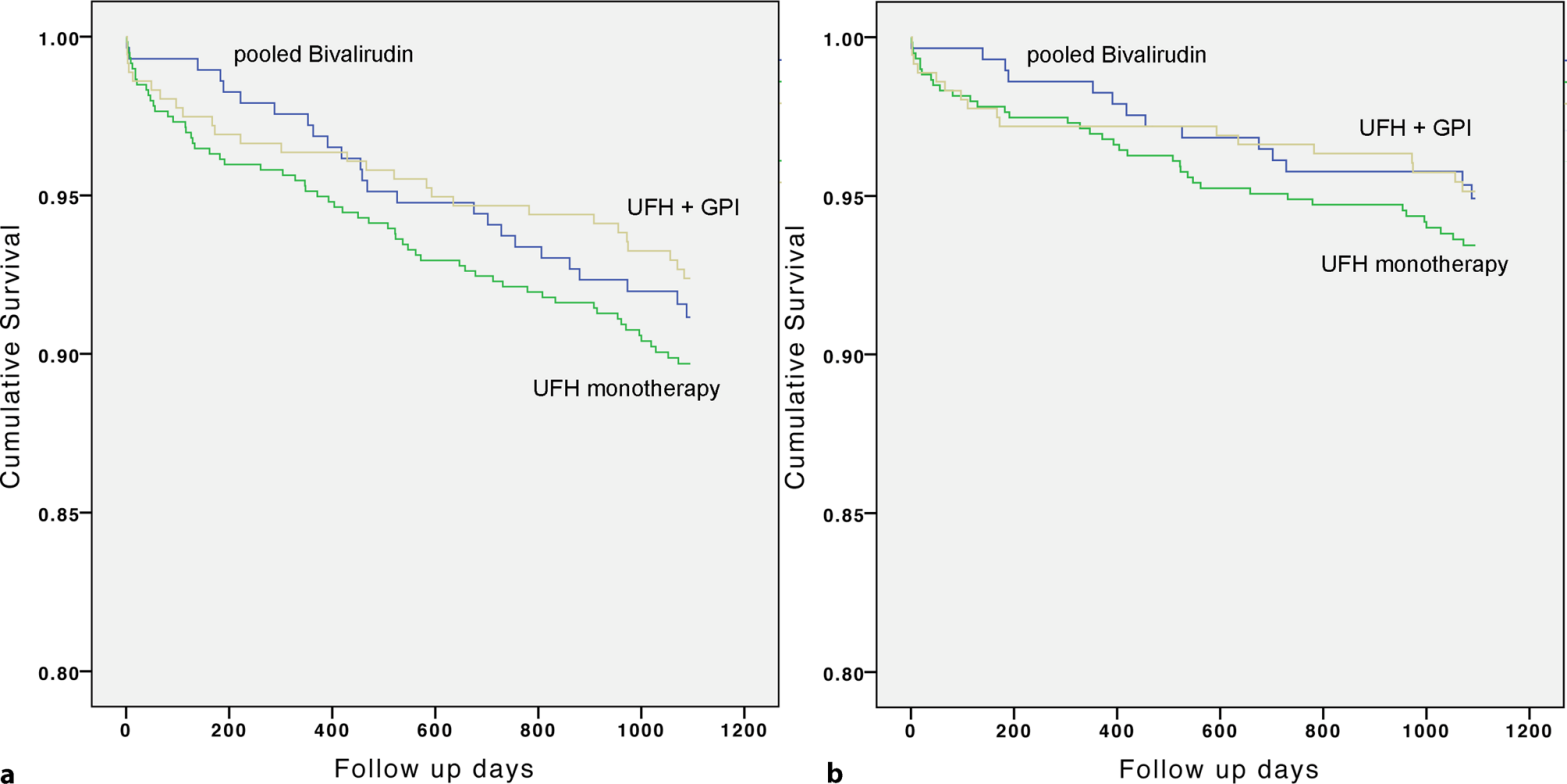
Kaplan-Meier plots for all-cause death (**a**) and cardiovascular death (**b**) after 3 years, stratified for antithrombotic treatment strategy. On propensity score adjusted Cox regression modelling, there were no significant differences between groups for both endpoints (Table 2)

After adjustment for confounders, similar mortality rates were observed for all comparisons. Univariate and multivariate analyses are presented in Table 2. Kaplan-Meier survival curves are shown in Fig. 1.

### Stent thrombosis

Acute or subacute stent thrombosis occurred in 10 (0.8 %) patients. In the pooled bivalirudin group 1 (0.4 %) definite, subacute event occurred. In the UFH monotherapy group, three definite and two probable events occurred (0.8 %) of which two were acute and three subacute. In the UFH + abciximab group, we observed two definite and two probable stent thromboses (1.1 %) of which one event was acute. The differences between groups were not statistically significant (*p* = 0.748) (Table 2).

### Bleeding outcome

**Fig. 2:**
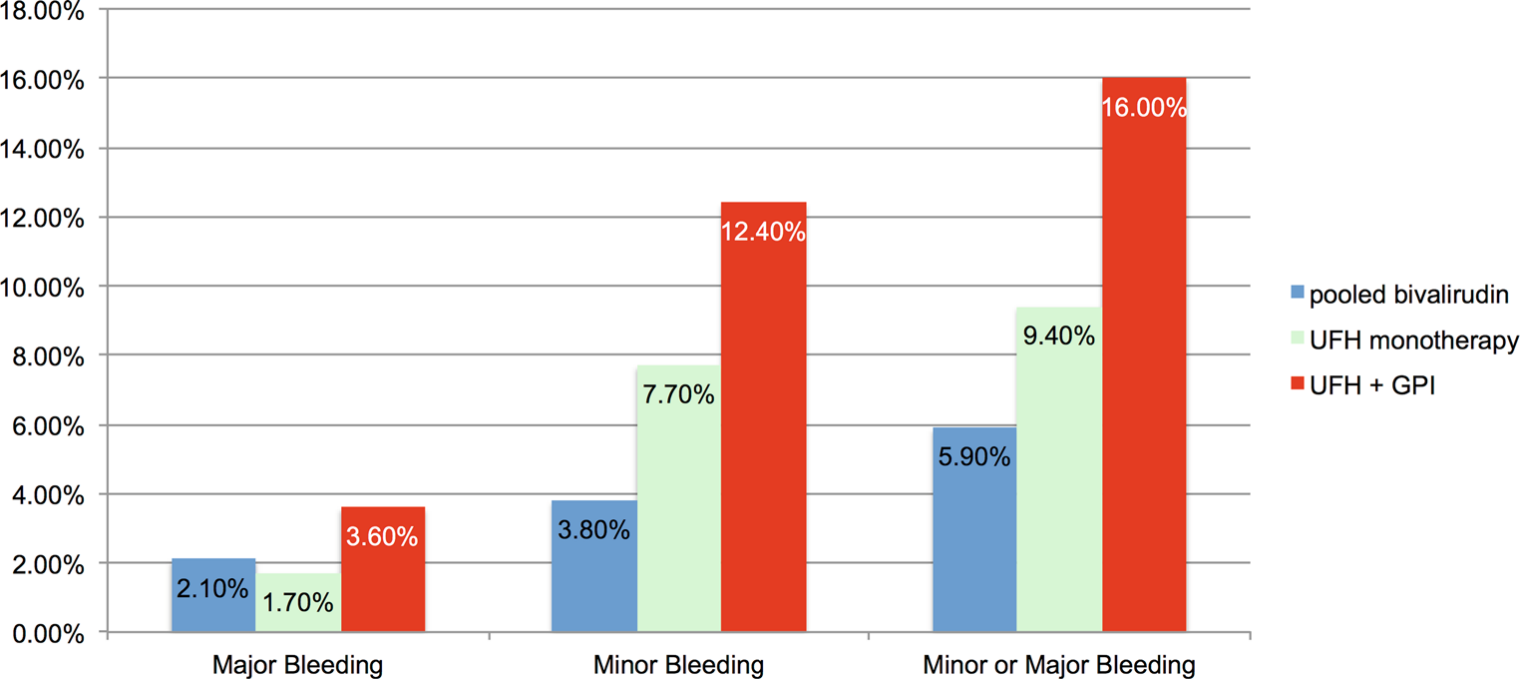
Rates of in-hospital bleeding stratified by anticoagulant treatment groups. Combined minor or major bleeding events were significantly lower comparing pooled bivalirudin vs. UFH + abciximab (OR 0.41, 95 % CI 0.22–0.78, *p* = 0.01), whereas bleeding rates were similar between bivalirudin vs. UFH alone (OR 0.82, 95 % CI 0.45–1.51, *p* = 0.53)

As shown in Fig. 2 composite rates of in-hospital TIMI minor or major bleeding were 5.9 % for the pooled bivalirudin group, whereas these bleeding rates were 9.4 % for the UFH monotherapy, 16 % for the UFH + abciximab group and 11.9 % for the pooled UFH group.

Univariate and multivariate analyses in the logistic regression model are presented in Table 3.

**Table 3:** Unadjusted and adjusted in-hospital minor or major bleeding rates

**Univariate outcomes**			
	*OR*	*95 % CI*	*p-value*
Pooled bivalirudin vs. UFH monotherapy	0.61	0.35; 1.06	0.08
Pooled bivalirudin vs. UFH +GPI	0.33	0.19; 0.58	<0.01
Pooled bivalirudin vs. pooled UFH	0.47	0.28; 0.79	<0.01
UFH monotherapy vs. UFH +GPI	0.55	0.37; 0.81	<0.01
**Adjusted outcomes**			
	*OR*	*95 % CI*	*p-value*
Pooled bivalirudin vs. UFH monotherapy	0.82	0.45; 1.51	0.53
Pooled bivalirudin vs. UFH +GPI	0.41	0.22; 0.78	0.01
Pooled bivalirudin vs. pooled UFH	0.62	0.36; 1.08	0.09
UFH monotherapy vs. UFH +GPI	0.65	0.43; 0.99	0.047

*UFH* unfractionated heparin, *GPI* glycoprotein IIb/IIIa inhibitor, *OR* odds ratio, *CI* confidence interval

**Fig. 3:**
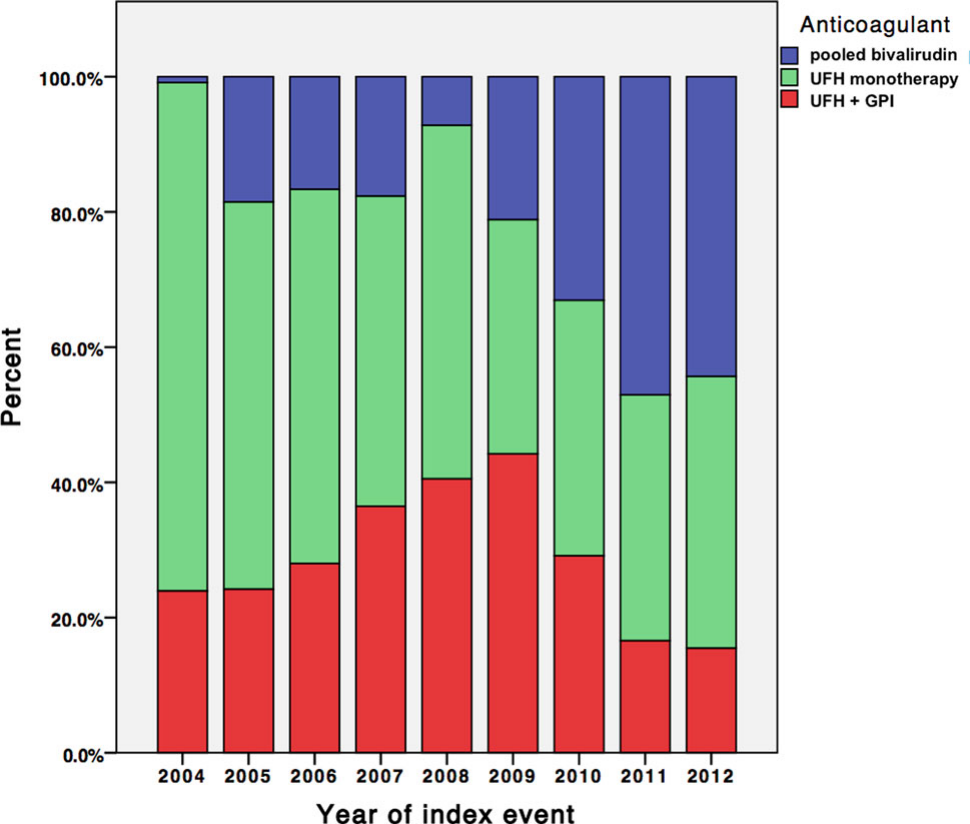
Utilization of the different antithrombotic strategies over time. Timely linked to the publication of the HORIZONS-AMI trial, there was a significant increase in bivalirudin utilization, whereas strategies including abciximab were less frequently used thereafter

After adjustment for confounding baseline variables bivalirudin use was associated with significantly lower rates in the composite endpoint of TIMI minor or major bleeding as compared to UFH + abciximab (5.9 % vs. 16 %, OR 0.41, 95 % CI 0.22–0.78, *p* = 0.01). Adjusted rates of bleeding were non-significantly lower for pooled bivalirudin vs. UFH monotherapy (5.9 % vs. 9.4 %, OR 0.82, 95 % CI 0.45–1.51, *p* = 0.53), and were reduced by 35 % comparing UFH alone to UFH + abciximab (9.4 % vs. 16 %, OR 0.65, 95 % CI 0.43–0.99, *p* = 0.047). Comparing the pooled bivalirudin vs. the pooled UFH group, there was a non-significant trend towards reduced bleeding rates (5.9 % vs. 11.9 %, OR 0.62, 95 % 0.36–1.08, *p* = 0.09). The year of treatment was accounted for in all adjusted analyses and the utilization of anticoagulants over time is presented in Fig. 3.

### Duration of hospitalization

**Fig. 4:**
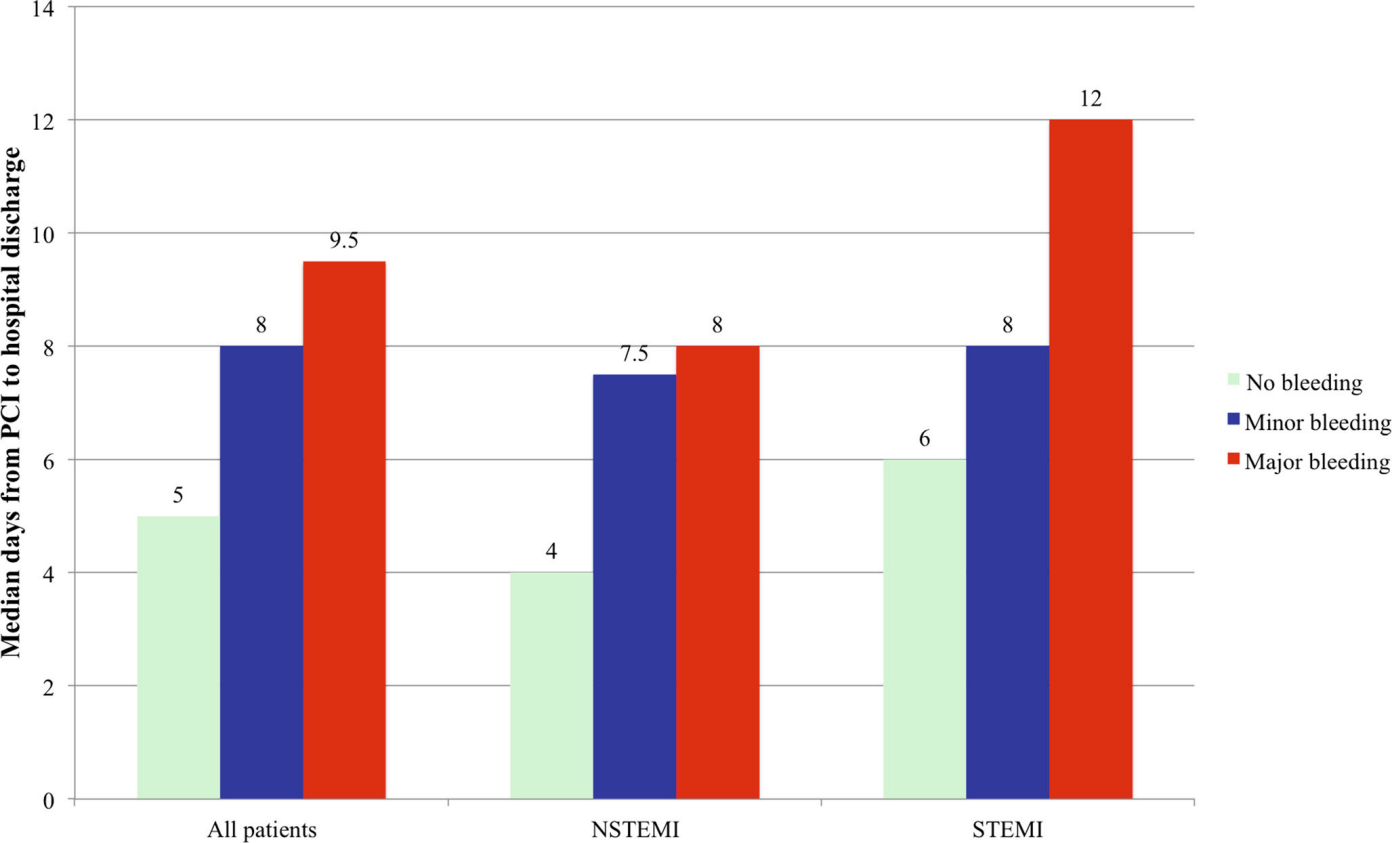
Median days from PCI to hospital discharge for the overall cohort and stratified by clinical presentation and bleeding severity. On adjustment for confounders, major and minor bleeding prolonged post-PCI hospital stay by 6.5 (95% CI 4.5–8.5, *p* < 0.01) and 3.2 (95 % CI 2.0–4.3, *p* < 0.01) days, respectively, compared to patients not experiencing a bleeding episode

Median days from PCI to hospital discharge are presented in Fig. 4. After adjustment for confounding baseline variables major and minor bleeding prolonged post-PCI hospital stay by 6.5 (95%CI 4.5–8.5, *p* < 0.01) and 3.2 (95 % CI 2.0–4.3, *p* < 0.01) days, respectively, as compared to patients without an in-hospital bleeding episode.

Median days from PCI to hospital discharge were 5 (Interquartile range [IQR] 2;7), 5 (IQR 2; 7) and 7 (IQR 5; 8) in the pooled bivalirudin, UFH monotherapy and UFH + abciximab groups, respectively. After adjustment for confounders days from PCI to discharge were similar between the pooled bivalirudin and UFH monotherapy groups (−0.8, 95 % CI −1.7 to −0.06, *p* = 0.07) but reduced by 1.5 (95 % CI −2.2 to −0.7, *p* < 0.01) days compared to UFH + abciximab.

## Discussion

In this retrospective analysis of a prospective registry, reflecting a real world situation, we were able to confirm the results of most randomized controlled trials in ACS patients undergoing invasive revascularization. Bivalirudin use was associated with a 59 % relative risk reduction in periprocedural combined TIMI minor or major bleeding rates compared to UFH +GPI, along with a reduced hospital stay after PCI. Bleeding rates were numerically but not statistically significantly lower comparing pooled bivalirudin vs. UFH alone or the pooled UFH group, whereas the rates of stent thrombosis, all-cause death and cardiovascular death were not statistically different between groups. Abciximab use increased significantly between 2006 and 2009 probably because of the recommendations issued by the American College of Cardiology/American Heart Association (ACC/AHA) guidelines on STEMI (2004) and NSTEMI (2007) as well as by the European Society of Cardiology (ESC) guidelines on the use of antiplatelet agents (2004) [14–16]. Moreover, timely linked to the publication of the HORIZONS-AMI trial in 2008, there was a significant increase in bivalirudin utilization, whereas strategies including abciximab were less recommended and also used thereafter [3]. This underlines the adherence to international guidelines at our center.

### Mortality outcome

Several earlier randomized trials conducted in the setting of moderate to high-risk NSTEMI (ACUITY and ISAR REACT 4) and STEMI (HORIZONS-AMI) revealed a positive net clinical benefit in favor of bivalirudin vs. UFH + GPI, primarily through the reduction of major bleeding events [3, 10, 11, 17]. The more recent EUROMAX trial investigated bivalirudin (with 12 % GPI use) vs. UFH (with 69 % GPI use) in a modern treatment setting for STEMI (50 % novel P2Y12 inhibitors) and also demonstrated a positive net clinical benefit in favor of bivalirudin. This benefit was mainly achieved through the reduction of major bleeding events, whereas cardiac death was not affected by the choice of the anticoagulant [7]. As the use of GPIs is usually low in a contemporary clinical setting, while radial access and novel P2Y_12_ receptor inhibitors are increasingly being used, the comparison vs. UFH monotherapy is particularly desirable [18]. The BRIGHT trial randomly assigned 2194 STEMI patients to bivalirudin with a 3‑h postprocedural infusion, UFH alone or UFH + tirofiban and reported no significant differences regarding major adverse cardiac or cerebral events at 30 days [8]. The recent MATRIX trial randomized 7213 ACS patients to either bivalirudin or UFH alone (only 0.2 % GPI use) and did not show a net improvement in adverse clinical events. Although cardiac death was reduced by 32 % (likely through reduction of major bleeding), this was counterbalanced by an excess in definite stent thrombosis [4]. In the single center HEAT-PPCI trial, also conducted in a modern setting (15 % GPI bail-out use, 90 % novel P2Y_12_ receptor inhibitors and over 80 % radial access) major adverse cardiovascular events were significantly increased by 52 % in the bivalirudin arm, with a fourfold risk of stent thrombosis and also an increase in bleeding hazards [9]. Meta-analyses provided conflicting results depending on the selection of these trials regarding reinfarction but showed no differences in mortality despite an increase in acute stent thrombosis in bivalirudin treated patients [19, 20]. In line with these findings we did not observe a mortality difference between groups, keeping in mind that statistical power was limited due to low sample size.

### Stent thrombosis

The rate of stent thrombosis was in the range of previously published data, also considering that 51 % of patients received bare metal stents [21]. We did not observe significant differences between groups; however, event rates were too low for statistical adjustment for confounders. In this setting there was no excess in acute stent thrombosis in patients treated with bivalirudin, opposed to data from previous large-scale trials [4, 7, 9].

### Bleeding outcomes

Our data are similar to the findings from the HORIZONS-AMI, EUROMAX, ACUITY, ISAR-REACT 4, and REPLACE-2 trials regarding significantly reduced bleeding hazards for bivalirudin vs. UFH + GPI [3, 7, 10, 11, 22]. As expected, we also observed lower bleeding rates when UFH monotherapy was compared to UFH + abciximab. It has to be noted that from a clinical point of view, comparisons against UFH + GPI are less relevant as recent guidelines recommend to restrict GPI use to bail-out situations [18, 23].

Data from prospective, randomized trials comparing bivalirudin and UFH monotherapies with respect to bleeding rates pointed in the same direction in both stable and ACS patients: [4, 8, 24]. In the recent BRIGHT and MATRIX trials, bleeding rates were significantly lower in favor of bivalirudin vs. UFH alone even when bivalirudin was continued following the procedure [4, 8]. Interestingly, no differences in major bleeding could be observed in the HEAT-PPCI trial [9] conducted in a modern setting with respect to antiplatelet agents, access site and GPI use. A potential explanation could be the prominent use of new P2Y12 receptor inhibitors that are known to be associated with significantly higher spontaneous bleeding rates [5, 25]. This was further confirmed by a post hoc analysis of EUROMAX demonstrating similar clinical outcome but higher per protocol major bleeding rates in patients treated with prasugrel or ticagrelor as compared to clopidogrel [26]. Although in-hospital bleeding rates in ACS patients undergoing PCI might also depend on the vascular access site and the majority of femoral access in our study might have influenced bleeding rates, a recent analysis has shown benefits for bivalirudin over UFH monotherapy and over UFH +GPI for both access sites [7].

### Hospital stay

The occurrence of TIMI minor and major bleeding had a substantial impact on post-PCI hospital stay in our analysis. As also described by others, major bleeding hazards accounted for an almost twofold prolongation of hospitalization in ACS patients [27]. Thus, preventing bleeding is important to preserve healthcare resource consumption, in addition to optimizing clinical outcomes.

### Limitations

The data from the present study were collected from a single center and in a non-randomized fashion as the treatment strategy was at the discretion of the treating interventional cardiologist; however, the 100 % follow-up rate strengthens the quality of data collection in this registry. Moreover, the number of patients in the bivalirudin arm was relatively low. This is mainly due to the fact that the majority of patients were included before publication of the recent NSTEMI and STEMI guidelines recommending bivalirudin monotherapy over UFH and UFH + GPI [28, 29]. Potential differences between treatment groups were existent but were accounted for in propensity score-adjusted models. Finally, additional safety and efficacy endpoints such as stroke, reinfarction, stent thrombosis, repeat revascularization, transfusion and thrombocytopenia, which may vary between the study regimens, are not available. Our findings might not apply to patients treated via a radial approach.

## Conclusion

This real world experience in patients presenting with ACS and undergoing PCI and stent implantation indicates that bivalirudin is clinically similar to UFH monotherapy but superior to UFH + abciximab with respect to the reduction of bleeding events, which in turn was associated with a shorter hospital stay. The rate of stent thrombosis as well as long-term clinical outcomes in terms of all-cause and cardiovascular mortality were statistically similar between groups.
